# A Comparative Study of Depression Score in Anesthesia vs Radiology Technicians

**DOI:** 10.1192/j.eurpsy.2025.1370

**Published:** 2025-08-26

**Authors:** M. A. Ghrab, I. Sellami, A. Haddar, A. Feki, M. Hajjaji, M. L. Masmoudi, K. Jmal Hammami

**Affiliations:** 1 Occupational medicine, University Hospital Hedi Chaker; 2 LR/18/ES-28, University of Sfax; 3 Rheumatology, University Hospital Hedi Chaker, Sfax, Tunisia

## Abstract

**Introduction:**

Healthcare workers are exposed to many psychological constraints, making them vulnerable to mental health issues, like depression. However, these constraints, in addition to other organizational and environmental exposures, vary between specialties and wards.

**Objectives:**

The aim of this study is to compare depression score in anesthesia technicians (AT) and radiology technicians (RT).

**Methods:**

We conducted a cross-sectional study among AT and RT in both University Hospitals in Sfax, Tunisia, between January and July 2024 during periodic health assessment visits. Sociodemographic and professional data were collected. The Patient-Health-Questionnaire-9 (PHQ-9) was used to assess signs of depression.

**Results:**

A total of 79 technicians participated in the study, with 60 AT and 19 RT. Their mean age was 46.4±7.6 years and six of them were males. Ten participants (12.7%) had a known psychiatric history. The mean seniority was 22.2±7.7 years. Sixty-two percent of the population had night shift work. The median PHQ-9 score was 7 interquartile range IQR [4;12]. Moderate to severe signs of depression were found in 32.9% of the population. Depression scores were significantly higher among RT with a median of 10 IQR [6;15] compared to a median of 7 IQR [2;11] among AT (p=0.04). PHQ-9 was not associated with age (p=0.15), sex (p=0.9) or seniority (p=0.06).

**Conclusions:**

Both AT and RT presented signs of depression. The difference of scores between the two groups stirs interests about the explaining factors. Further studies detailing different occupational constraints and exposure are needed.

**Disclosure of Interest:**

None Declared

